# Cybersecurity threats and preparedness: Implications for dental schools

**DOI:** 10.1002/jdd.13758

**Published:** 2024-10-27

**Authors:** Romesh P. Nalliah, Suvesha Praveen, Trishul V. Allareddy, Praveenkumar Gajendrareddy, Min Kyeong Lee, Maysaa Oubaidin, Veerasathpurush Allareddy

**Affiliations:** ^1^ Office of Patient Services University of Michigan School of Dentistry Ann Arbor Michigan USA; ^2^ Department of Orthodontics University of Illinois at Chicago College of Dentistry Chicago Illinois USA; ^3^ Department of Oral Pathology, Radiology and Medicine The University of Iowa – College of Dentistry Iowa City Iowa USA; ^4^ Oral and Maxillofacial Radiology The University of Iowa – College of Dentistry Iowa City Iowa USA; ^5^ Department of Periodontics University of Illinois at Chicago College of Dentistry Chicago Illinois USA

**Keywords:** behavioral sciences, cost, dental care access, dental faculty, economics of dental education, education, ethics, faculty, health care access, health care systems, patient affairs, patient care management, patient safety, professional interest, professional responsibility

## Abstract

**Background:**

Cybersecurity threats are a growing concern in healthcare, where digital systems now underpin patient care, financial management, and educational operations. A cybersecurity breach in a Dental school environment can have widespread consequences to the mission of the school—patient care, research, education and service. For dental school administrators, these risks highlight the necessity of robust cybersecurity measures. For student learners, the impact may include interruptions to their education. For patients, it could mean compromised personal data and reduced access to clinical care.

**Results & Conclusion:**

While many sectors have responded to increasing cyber threats by enhancing their defenses, healthcare and dental schools, often lag in implementing necessary protections. This emphasizes the need for proactive measures, such as regular system audits, advanced encryption methods, and ongoing cybersecurity training for administrators and students alike, to mitigate future risks and safeguard institutional integrity.

## 1 CONTEXT

In August of 2023, one of the dental schools in the Midwest region was a victim of a major cybersecurity attack.^1^ The personal data of School of Dentistry patients, University Health Service patients, some students, applicants, alumni, donors, employees, contractors, and research participants was breached.^2^ After this experience, the authors of this article wanted to disseminate information about the experience to enable other dental schools to be well prepared for future threats. Cybersecurity threats can take many forms, including data breaches, ransomware, phishing, and malware attacks. These attacks target sensitive medical and financial information, potentially causing widespread disruption in dental schools.

The increasing prevalence of cybersecurity attacks has profoundly impacted various sectors. These incidents have caused substantial financial losses and jeopardized sensitive personal information and national security. The healthcare sector has also been a significant target. In 2020, a ransomware attack on Universal Health Services, a major healthcare provider in the United States, disrupted operations across 400 facilities, delaying critical medical procedures and patient care.^3^ The attack cost these companies over $67 million, demonstrating the severe financial and operational impacts of cybersecurity breaches in healthcare.^4^ This disruption lasted three weeks, illustrates the far‐reaching consequences of cybersecurity attacks, and emphasizes the need for enhanced security protocols and proactive measures to protect against future threats.

In the context of dental schools in the United States, integrating digital and online systems has become deeply embedded in clinical, educational, and research activities.^5^ These systems manage everything from medical and financial records to e‐prescribing and educational software. Given the comprehensive digital nature of these institutions, the ramifications of such attacks are profound and multifaceted. While cybersecurity attacks pose significant threats to various sectors, a systematic review showed that healthcare is particularly vulnerable because it has not kept up with threats.^6^

## 2 TYPES OF CYBER THREATS

Hospitals and often, by their extension, dental schools and or practices face a variety of cyber threats, with some of the most common attack vectors include:

***Ransomware***: Ransomware attacks are one of the most prevalent threats in healthcare. Attackers deploy malware to encrypt hospital data and demand a ransom payment, often in cryptocurrency, to provide the decryption key. High‐profile incidents, such as the 2017 WannaCry attack, demonstrated the devastating impact ransomware can have on healthcare systems worldwide.^7^
***Phishing***: Phishing attacks involve tricking employees into revealing sensitive/personal/confidential information or downloading malware through deceptive emails or messages. These attacks often target hospital staff with emails that appear to be from trusted sources, leading to compromised login credentials or the installation of malicious software.^8^
***Distributed Denial of Service Attacks***: Distributed Denial of Service attacks overwhelm hospital servers with traffic, rendering systems inaccessible to legitimate users. This attack can disrupt hospital operations, affecting everything from patient admissions to electronic health records systems.^9^
***Insider Threats***: Insider threats involve individuals (such as employees or contractors) within the organization, who misuse their access to sensitive information. This can be intentional, such as data theft, or accidental, due to negligent information handling.^10^
***Vulnerabilities in Medical Devices and Systems***: Many medical devices and systems, such as equipment for imaging patients, are connected to dental school networks but may lack robust security measures. Exploiting vulnerabilities in these devices can give attackers access to broader hospital or dental school systems.^11^
***Open network ports***: Open network ports in organizations can pose significant cybersecurity risks. Ports are gateways through which data enters and leaves a network. While some must remain open for essential services (like web servers or email), others can become entry points for cyber attackers if not properly secured.^12^

## 3 IMPACT ON CLINICAL AND EDUCATIONAL ACTIVITIES

Cyber attacks can catastrophically impact clinical and educational activities in a dental school. A few of them are summarized below:

***Disruption of Patient Care***: A cybersecurity attack could lead to the immediate suspension of clinical activities or risks to patient safety if care delivery continues.^13^ Dental health records, which interface with finance tools, e‐prescribing systems, and educational software, are critical for patient care. A breach could render these health records inaccessible, delaying treatments, threatening patient safety, and disrupting patient schedules.^14^
***Data Loss and Integrity***: Compromised systems may lead to the loss or alteration of patient data. This can affect everything from health history and radiology images to treatment plans. Restoring data integrity is often a time‐consuming and complex process.^15^
***Financial Chaos***: Integrated finance tools are vulnerable to cybersecurity attacks. Financial transactions, insurance claims, and billing processes could be halted, causing financial strain on the institution and its patients.^16^
***E‐prescribing Interruption***: E‐prescribing systems, crucial for the timely administration of medications, can be rendered inoperative. This affects patient treatment and adherence to legal and medical protocols.^17^
***Educational Disruption***: Although there is no evidence comparing cybersecurity in healthcare to education, the latter may be even less prepared than the healthcare system. With the growth of online education and the expansion of the reliance on educational software, the education system is very vulnerable to cyberattacks.^18^ Cyberattacks can halt safe access to educational software and digital learning platforms, disrupting the educational process for both undergraduate and graduate students. Clinical training, which relies heavily on digital records and simulations, can also be affected.
***Loss of Trust and Reputation***: Repeated or severe cyberattacks can erode trust in technology and an institution.^19^ In a dental school environment, patients may seek care elsewhere, and prospective students might choose schools perceived as more secure.
***Regulatory and Legal Repercussions***: Healthcare clinics and dental schools could face regulatory scrutiny and legal actions due to the breach of protected health information. Compliance with HIPAA and other regulations becomes a critical issue.^20^ Since 2015, hacking and cybersecurity incidents have become the primary cause of data breaches in the United States—surpassing theft, improper disposal, and unauthorized access or disclosure.^20^
***Ongoing Financial Burden***: Beyond immediate financial chaos, long‐term costs include legal fees, regulatory fines, and investments in improved cybersecurity infrastructure. These financial burdens can impact the institution's budget and resources available for educational programs.^13^

## 4 MITIGATION STRATEGIES

In a time where technological advancements have changed almost every aspect of our lives, the sector of dentistry isn't an exception.^21^ Online resources have streamlined administrative responsibilities, progressed in‐person and online care, and helped make better dental practices. However, this transformation to a dependency on the internet has added new vulnerabilities, leaving dental places susceptible to cyber‐attacks. Understanding the motivations and outcomes of such attacks is necessary for safeguarding sensitive patient data and maintaining the integrity of dental practices.^22^

In the modern digital age, cybersecurity breaches pose significant threats to educational institutions, including dental schools. Dental schools must prioritize employee education and awareness programs to inform staff members about cybersecurity standards and potential risks.^23^ Protecting sensitive data and maintaining the integrity of institutional systems are paramount. To effectively mitigate these risks, dental schools can implement several strategic measures. A comprehensive cybersecurity framework is the cornerstone of an effective defense against cyber threats. This framework should encompass regular security assessments to identify vulnerabilities, deploying intrusion detection systems to monitor and respond to suspicious activities, and implementing updated antivirus software to protect against malware.^24^ These elements collectively form a strong barrier against potential breaches, ensuring the institution is well‐prepared to counteract cyber threats.

Regular education about vigilance and identifying threats for the dental school community is another critical component in the fight against cyber threats. Faculty, staff, and students must be regularly trained on cybersecurity best practices to prevent phishing attacks and other common threats (Table 1). Perhaps it can be incorporated into other mandatory training like compliance and infection control. Experts emphasize the importance of continuous training sessions, which enhance the overall awareness and preparedness of the dental school community.^25^ Institutions can significantly reduce the likelihood of successful cyber attacks by fostering a culture of vigilance and informed behavior.

**TABLE 1:** Employee training and awareness.

Type of training	Outcomes
Phishing Awareness	Educating staff on recognizing phishing attempts, which are common entry points for cyberattacks
Safe Browsing Practices	Training on avoiding unsafe websites and downloads that could introduce malware
Security Protocols Compliance	Ensuring staff understand and comply with the organization's cybersecurity policies, including password management and secure data handling

Encrypting sensitive data and maintaining a regular backup protocol is essential to mitigate the damage caused by cybersecurity breaches. Encryption ensures that without the decryption key, the intercepted data remains unreadable. Regular backups, stored offsite and in secure locations, guarantee that data can be restored in the event of an attack. These measures safeguard institutional data and ensure continuity of operations.^26^

Implementing multi‐factor authentication (MFA) for access to sensitive systems adds an additional layer of security beyond traditional passwords. MFA requires users to provide several forms of verification (for example a temporary code sent to their mobile device or on an application after entering their password). This approach significantly reduces the risk of unauthorized access, as it is much harder for attackers to compromise multiple authentication factors simultaneously.^27^

Developing and timely updating of an incident response plan is crucial for ensuring a swift and effective reaction to cybersecurity breaches. An incident response plan should outline clear communication strategies, data recovery processes, and specific roles and responsibilities for handling an attack. A technical report by Killcrece and colleagues stresses the importance of such a plan in minimizing the impact of breaches and facilitating a coordinated response that can quickly restore normal operations.^28^

Partnering with cybersecurity experts and firms provides dental schools with advanced protection and insights into emerging threats. It may become standard practice to have one or more cybersecurity experts on the dental informatics team, and regular third‐party audits can help identify potential vulnerabilities and recommend effective countermeasures. Based on existing evidence, we also recommend collaboration in maintaining robust defenses and staying ahead of evolving cyber threats.^29^

Regular security audits and assessments are fundamental to identifying and addressing vulnerabilities within any healthcare provider's practice.^30^ These audits should encompass various activities listed in Table 2. Protecting the confidentiality and integrity of data is crucial in healthcare settings.^31^, ^32^ Key measures include Encryption (Data cannot be read without the decryption key) and Access Controls (MFA and ensuring that access to data is based on the principle of least privilege).

**TABLE 2:** Security audits and assessments.

Type of audit	Assessment
Penetration Testing	This involves simulating cyberattacks to evaluate the security posture of systems and identify exploitable weaknesses.
Vulnerability Scanning	Automated tools can scan networks and systems for known vulnerabilities, ensuring that these are addressed promptly.
Access Controls Review	This includes reviewing and refining user permissions to ensure that only authorized individuals have access to sensitive data and systems.
Data Handling Practices	Ensuring that data is handled in compliance with security protocols and regulatory requirements

Dental and medical devices are especially vulnerable to cyberattacks as they are often connected to the network in practice and lack robust security measures.^32^ Measures to minimize risks are provided in Table 3.^33^

**TABLE 3:** Measures to minimize risks.

Measures	Description
Regular Updates and Patching	Ensuring that all devices and systems are regularly updated with the latest security patches
Network Segmentation	Dividing the network into segments to limit the spread of malware and restrict access to sensitive data
Device Authentication	Implementing strict authentication measures for devices accessing the network

Compliance with healthcare‐specific regulations and cybersecurity frameworks is crucial for maintaining high‐security standards, patient confidentiality, and compliance with privacy regulations such as the Health Insurance Portability and Accountability Act in the United States or the General Data Protection Regulation in Europe.^34^, ^35^

## 5 CONCLUSIONS

In conclusion, cyber‐attacks are a grave threat to the security of dental schools. The potential impact of cybersecurity attacks on dental school activities is profound, affecting clinical operations, educational continuity, and stakeholder trust. By understanding the immediate and long‐term implications, university‐based dental schools in the United States can implement effective prevention and mitigation strategies. Robust cybersecurity measures, regular training, and an agile response plan are essential to safeguard these institutions against future threats.
